# A differential risk assessment and decision model for Transarterial chemoembolization in hepatocellular carcinoma based on hepatic function

**DOI:** 10.1186/s12885-020-06975-2

**Published:** 2020-06-01

**Authors:** Joon Yeul Nam, A Reum Choe, Dong Hyun Sinn, Jeong-Hoon Lee, Hwi Young Kim, Su Jong Yu, Yoon Jun Kim, Jung-Hwan Yoon, Jeong Min Lee, Jin Wook Chung, Sun Young Choi, Jeong Kyong Lee, Seung Yon Baek, Hye Ah Lee, Tae Hun Kim, Kwon Yoo

**Affiliations:** 1 Department of Internal Medicine, College of Medicine, Ewha Womans University, 1071, Anyangcheon-ro, Yangcheon-gu, Seoul, 07985 Republic of Korea; 2 Department of Internal Medicine and Liver Research Institute, Seoul National University College of Medicine, 101, Daehak-ro, Jongno-gu, Seoul, 03080 Republic of Korea; 3 Department of Internal Medicine, Samsung Medical Center, Sungkyunkwan University School of Medicine, Seoul, Republic of Korea; 4 Department of Radiology, Seoul National University College of Medicine, Seoul, Republic of Korea; 5 Department of Radiology, College of Medicine, Ewha Womans University, Seoul, Republic of Korea; 6 Clinical Trial Center, Ewha Womans University Mokdong Hospital, Seoul, Republic of Korea

**Keywords:** Hepatocellular carcinoma, Transarterial chemoembolization, Child-Pugh classification, Risk prediction model

## Abstract

**Background:**

The decision of transarterial chemoembolization (TACE) initiation and/or repetition remains challenging in patients with unresectable hepatocellular carcinoma (HCC). The aim was to develop a prognostic scoring system to guide TACE initiation/repetition.

**Methods:**

A total of 597 consecutive patients who underwent TACE as their initial treatment for unresectable HCC were included. We derived a prediction model using independent risk factors for overall survival (OS), which was externally validated in an independent cohort (*n* = 739).

**Results:**

Independent risk factors of OS included Albumin-bilirubin (ALBI) grade, maximal tumor size, alpha-fetoprotein, and tumor response to initial TACE, which were used to develop a scoring system (“ASAR”). C-index values for OS were 0.733 (95% confidence interval [CI] = 0.570–0.871) in the derivation, 0.700 (95% CI = 0.445–0.905) in the internal validation, and 0.680 (95% CI = 0.652–0.707) in the external validation, respectively. Patients with ASAR< 4 showed significantly longer OS than patients with ASAR≥4 in all three datasets (all *P* < 0.001). Among Child-Pugh class B patients, a modified model without TACE response, i.e., “ASA(R)”, discriminated OS with a c-index of 0.788 (95% CI, 0.703–0.876) in the derivation, and 0.745 (95% CI, 0.646–0.862) in the internal validation, and 0.670 (95% CI, 0.605–0.725) in the external validation, respectively. Child-Pugh B patients with ASA(R) < 4 showed significantly longer OS than patients with ASA(R) ≥ 4 in all three datasets (all *P* < 0.001).

**Conclusions:**

ASAR provides refined prognostication for repetition of TACE in patients with unresectable HCC. For Child-Pugh class B patients, a modified model with baseline factors might guide TACE initiation.

## Background

Hepatocellular carcinoma (HCC) remains the fifth most common malignancy and is the second most common cause of cancer-related mortality worldwide [1]. Because many patients are still diagnosed with unresectable diseases, transarterial chemoembolization (TACE) is the standard treatment for such patients in the absence of macroscopic vascular invasion or extrahepatic spread, which comprises mostly intermediate stage or Barcelona Clinic Liver Cancer (BCLC) stage B [2, 3]. However, the survival benefit of TACE is not universal even in patients with same tumor stage, mainly because of the heterogeneity of the tumor burden and/or the hepatic functional reserve [4, 5].

Several strategies for patient selection in terms of initiation or repetition of TACE have been suggested, mostly combining tumor factors and hepatic functional reserve: for example, hepatoma arterial-embolization prognostic (HAP) score (Supplementary material), BCLC B sub-classification, Assessment for Retreatment with TACE (ART) score, etc. [5–8] However, some subsequent studies on these strategies have reported mixed results [5, 9–11]. Given that the decision of TACE as an initial treatment primarily depends on hepatic functional reserve for patients with TACE-treatable tumors, TACE is mostly reserved for those with Child-Pugh class A or for highly selected Child-Pugh class B patients without decompensation [2, 3]. For patients with Child-Pugh class A undergoing repeated sessions of TACE, it is important to predict TACE failure or refractoriness early enough to shift toward systemic treatment at an appropriate timing. However, for Child-Pugh class B patients, proper patient selection for the initiation of TACE seems relevant to prevent further deterioration of hepatic function and the resulting worsening in patient survival. Thus, a differentiated approach for patient selection based on hepatic function is required for patients who are potential candidates for TACE.

Hepatic functional reserve has traditionally been assessed using the Child-Pugh system [12]. However, the Child-Pugh system has several limitations such as inclusion of subjective variables (ascites and encephalopathy grade), absence of weighting for each variable, and changeable cut-off [13]. In this regard, the albumin-bilirubin (ALBI) grade (Supplementary material) was developed to assess hepatic function of HCC patients, using only objective variables (combination of serum albumin and bilirubin), and has shown to be useful in stratifying HCC patients across different stages [14–16]. In addition to hepatic functional assessment, tumor burden should also be included in the decision making of initiation or repetition of TACE.

Thus, the present study aimed to develop and validate a prognostic scoring system using a combination of hepatic function and tumor factors, and investigate the outcomes according to the scores to shed light on patient selection for the initiation or repetition of TACE based on the presence or absence of impaired hepatic functional reserve.

## Methods

### Patients

**Fig. 1:**
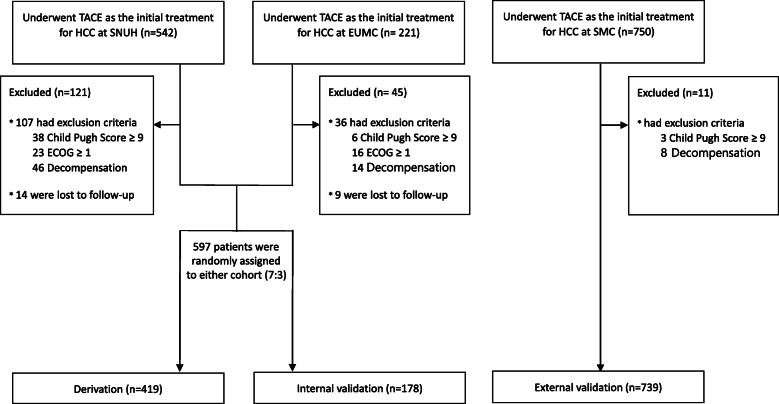
Consort diagram. A total of 763 HCC patients who received TACE as initial treatment for unresectable HCC were enrolled from two large-volume university hospitals. Of these, 166 patients were excluded and 597 patients were enrolled for analysis. A total of 739 HCC patients from independent university hospital were enrolled to validate the model externally

In this multi-center cohort study, eligible HCC patients were evaluated for eligibility from three large-volume university hospitals in South Korea (i.e., Seoul National University Hospital [SNUH], Ewha Womans University Medical Center [EUMC], and Samsung Medical Center [SMC]). We included consecutive patients meeting all the following inclusion criteria: 1) the patients with unresectable HCC of BCLC A or B; 2) the patients who received conventional TACE as their initial treatment; 3) the patients aged ≥18 years. The diagnosis of HCC was based on histological examination or clinicoradiological criteria according to international guidelines [2, 3]. Among a total of 763 consecutive HCC patients from SNUH (between January 2012 and April 2014, *n* = 542) and EUMC (between January 2011 and December 2015, *n* = 221) who were considered eligible, 166 patients (*n* = 121 in SNUH and *n* = 45 in EUMC) were excluded because of the following reasons: poor performance state (ECOG≥1, *n* = 39); Child-Pugh class B9 or C (*n* = 44), current or previous features of decompensation (i.e., uncontrolled ascites, variceal hemorrhage, or hepatic encephalopathy; *n* = 60), and follow-up loss (*n* = 23). Among these patients, Thus, 597 patients were enrolled for analysis, and were randomly assigned to either the derivation (*n* = 419) or the internal validation set (*n* = 178) stratified by age and sex at a 7:3 ratio (Fig. 1). For an independent cohort for external validation, 750 HCC patients who met the abovementioned criteria were evaluated for eligibility from SMC (between January 2007 and December 2012). Of these, 11 patients were excluded because of history of decompensation or Child-Pugh score of 9 or higher. Finally, 739 patients from SMC were enrolled for external validation.

The present study was approved by the institutional review boards of the three participating institutions and was conducted following the ethical guidelines of the World Medical Association Declaration of Helsinki. Informed patient consent was waived by the institutional review board of each institution owing to the retrospective nature of the study.

### TACE procedure and treatment schedule

TACE procedures were performed using the superselective method by experienced interventional radiologists (over 10 years of experience) in study hospitals [17]. The principles of TACE procedures were largely similar between three institutions in terms of superselectivity, choice of chemotherapeutic agents and treatment schedule as described elsewhere [18, 19]. Briefly, an arterial catheter was inserted into the femoral artery using the Seldinger technique and elective angiography of the celiac axis was performed. Then the catheter was advanced into the desired hepatic artery branch. Tumor-feeding vessels were superselected whenever possible, and a suspension containing 20–60 mg of doxorubicin hydrochloride (ADM, Dong-A Pharmacy, Seoul, Korea) and 2–20 mL of iodized oil (Lipiodol, Guerbet, Aulnay-sous-Bois, France) with absorbable gelatin sponge particles (Gelfoam, Upjohn, Kalamazoo, MI) was infused through a catheter (5-Fr) or a microcatheter (2.8- or 3-Fr) placed in the tumor-feeding arteries. The dosages of doxorubicin and iodized oil and the use of gelatin sponge particles were determined for each patient based on tumor burden, tumor characteristics, and hepatic functional reserve [20, 21]. Repeated TACE treatments were considered if residual or newly developed tumors were detected on dynamic computed tomography (CT) or magnetic resonance imaging (MRI) undertaken 4–6 weeks following each TACE session and were performed on an “on-demand” basis depending on individual tumor response and hepatic functional reserve.

### Outcomes and assessments

The primary outcome was overall survival (OS), which was measured from the date of HCC diagnosis to the date of death from any cause. Survival data of the enrolled patients were obtained from the national statistical data provided by the Korean Ministry of Government Administration and Home Affairs. The data cut-off date was November 30, 2017. The secondary outcome assessed was tumor response. Tumor responses were evaluated after every TACE session with the modified Response Evaluation Criteria in Solid Tumors criteria [22]. The efficacy of the performed TACE was determined by evaluating the pattern of iodized oil retention in the target lesions as an indicator of tumor necrosis [23]. Iodized oil retention was considered as compact on imaging when the contrast medium was well scattered throughout all viable target lesions; otherwise, it was regarded as non-compact uptake [24]. Patients without residual viable tumor after TACE were followed-up with dynamic CT or MRI every 8–12 weeks. All scans were reviewed by two independent radiologists with > 10 years of experience who were unaware of the group assignment. In cases of discordance, an additional third independent experienced radiologist reviewed the images and a consensus was reached among the three.

### Statistical analysis

Baseline characteristics were presented as mean ± standard deviation for normally distributed continuous variables and median with interquartile ranges (IQRs) for continuous variables with a skewed distribution. Discrete variables were summarized by the number of subjects with percentages. To compare baseline characteristics between groups, we used the Student’s t-test or Mann-Whitney U test, as appropriate. Distribution of categorical variables was compared using the chi-square and Fisher’s exact test. OS was calculated as the time from HCC diagnosis until death from any cause. Survival analysis was performed using the Kaplan-Meier analysis and the log-rank test was used to compare between groups. Tumor- and hepatic function-related prognostic factors for OS were explored using the Cox proportional-hazards regression analysis. Based on the results of the univariate analyses, factors with a significant difference (*P <* 0.05) were included in the multivariate model for development of a prediction model. A prediction model for OS was developed using relevant parameters identified by forward stepwise selection. A model with the minimum Akaike information criterion value was selected, which rewarded the goodness-of-fit of the model. Basically, we performed a proportional-hazards hypothesis for the selected models. The proportional-hazards hypothesis was checked via the Schoenfeld residuals method. The predictive ability was evaluated by using concordance (c)-statistics for discrimination function and Hosmer-Lemeshow test for calibration function. These were performed after excluding the variables sequentially to develop the simplest model out of candidate models developed. When the result of the Hosmer-Lemeshow test was satisfied (*P* > 0.05) and the c-statistics was 0.7 or more, the difference in c-statistics between the models was compared and the simplest model was selected if there was no statistically significant difference. To obtain the optimism corrected value of the c-statistics, we applied 100-times bootstraps. In the final model, a relative score of each risk factor was assigned based on the estimated coefficient value, and the risk score for prediction of OS was calculated in each subject. Based on the results of the pairwise log-rank test, the patients were divided into subgroups by the OS score and survival curves were compared between the subgroups. Internal and external validation of the developed prediction model was conducted, and sensitivity analyses were performed under various conditions (e.g., among patients with Child-Pugh class A or B). A two-sided *P <* 0.05 was considered statistically significant. All statistical analysis was performed using SAS ver. 9.4 software (SAS Institute, Cary, NC).

## Results

### Baseline patient characteristics

Table 1 summarizes the baseline characteristics of the overall included patients. The median age was 63 in SNUH, 69 in EUMC, and 63 years in SMC. The proportion of male was 79.1% in SNUH, 80.1% in EUMC, and 78.9% in SMC. The etiology of the underlying liver disease was mostly viral. The number tumors over 3 were found in 72 (17.1%) patients of SNUH, 32 (18.2%) of EUMC, and 120 (16.2%) of SMC. The maximal tumor diameter over 5 cm was found in 113 (26.8%) of SNUH, 57 (32.4%) of EUMC, and 272 (36.8%) of SMC. The median alpha-fetoprotein (AFP) level ≥ 200 ng/mL was found in 89 (21.1%) of SNUH, 49 (28.5%) of EUMC, and 194 (26.3%) of SMC. The patient with Child-Pugh class B was 85 (20.2%) in SMUH, 33 (18.8%) in EUMC, and 101 (13.7%) in SMC. The HAP score D were found in 24 patients (5.7%) in SNUH, 23 (13.1%) in EUMC, and 50 (6.8%) in SMC, respectively. ALBI grades 3 were 17 patients (4.0%) in SNUH, 13 (7.4%) in EUMC, and 15 (2.0%) in SMC (Table 1). The time intervals between the diagnosis of HCC and the initial TACE were 2.0 weeks (1.8–3.0) in SNU cohort and 2.0 weeks (1.0–2.8) in EUMC cohort (*P* = 0.115), suggesting that there was no significant time delay between the diagnosis and the initial treatment in both cohorts.

**Table 1:** Baseline characteristics of the entire cohort

Variables	SNUH (***n*** = 421)	EUMC (***n*** = 176)	SMC (n = 739)	***P***
Sex				
Male	333 (79.1)	141 (80.1)	583 (78.9)	0.938
Female	88 (20.9)	35 (19.9)	156 (21.1)	
Age (year)	63 (54–71)	69 (59–78)	63 (56–70)	< 0.001
Etiology				
HBV	265 (63.0)	121 (74.6)	517 (70.0)	0.005
HCV	56 (13.3)	26 (16.0)	97 (13.1)	
alcohol	45 (10.7)	15 (9.4)	67 (9.0)	
Cirrhosis				
Yes	336 (79.8%)	134 (76.1%)	443 (60.0)	< 0.001
Child-Pugh class^a^				
A	336 (79.8)	143 (81.3)	638 (86.3)	0.010
B	85 (20.2)	33 (18.8)	101 (13.7)	
BCLC stage				
A	168 (39.9)	69 (39.2)	500 (67.7)	< 0.001
B	253 (60.1)	107 (60.8)	239 (32.3)	
ALBI grade				
1	159 (37.8)	45 (25.6)	339 (45.9)	< 0.001
2	245 (58.2)	118 (67.0)	385 (52.1)	
3	17 (4.0)	13 (7.4)	15 (2.0)	
HAP score				
A	157 (37.3)	58 (33.0)	233 (31.5)	0.016
B	134 (31.8)	47 (26.7)	252 (34.1)	
C	106 (25.2)	48 (27.3)	204 (27.6)	
D	24 (5.7)	23 (13.1)	50 (6.8)	
Modified HAP score				
A	211 (50.1)	71 (40.4)	98 (13.3)	< 0.001
B	150 (35.6)	67 (38.1)	259 (35.0)	
C	55 (13.1)	29 (16.5)	235 (31.8)	
D	5 (1.2)	9 (5.1)	147 (19.9)	
Tumor number				
≤3	349 (82.9)	143 (81.7)	619 (82.8)	0.715
> 3	72 (17.1)	32 (18.2)	120 (16.2)	
Tumor size (cm)				
< 3	179 (42.5)	68 (38.6)	304 (41.1)	< 0.001
3–5	129 (30.6)	51 (29.0)	163 (22.1)	
≥5	113 (26.8)	57 (32.4)	272 (36.8)	
Hemoglobin (g/dL)	13.0 (11.8–14.3)	13.2 (11.3–14.5)	13.8 (12.7–14.8)	< 0.001
WBC (×10^3^/μL)	5.1 (3.9–6.3)	5.1 (3.9–6.3)	5.1 (4.0–6.6)	0.430
Platelet (× 10^3^/μL)	120 (81–165)	132 (91–179)	129 (87–175)	0.406
Prothrombin time (INR)	1.1 (1.0–1.2)	1.1 (1.1–1.2)	1.1 (1.1–1.2)	< 0.001
Creatinine (mg/dL)	0.8 (0.7–1.0)	0.9 (0.8–1.1)	0.9 (0.8–1.0)	< 0.001
Sodium (mEq/L)	141 (139–142)	139 (137–141)	140 (138–142)	< 0.001
AST (mg/dL)	42 (29–64)	44 (33–63.5)	49 (34–72)	0.089
ALT (mg/dL)	34 (22–54)	33 (23–53.5)	38 (24–58)	0.322
Total bilirubin (mg/dL)	0.8 (0.6–1.2)	0.8 (0.6–1.1)	0.8 (0.6–1.1)	0.487
Albumin (g/dl)	3.8 (3.4–4.1)	3.7 (3.4–4.0)	3.9 (3.5–4.2)	0.233
Alpha-fetoprotein (ng/mL)				
≥200	89 (21.1)	49 (28.5)	194 (26.3)	0.084
< 200	332 (78.9)	123 (71.5)	545 (73.4)	
NLR	1.85 (1.3–2.6)	1.90 (1.3–3.1)	1.6 (1.2–2.2)	< 0.001
No. of TACE sessions	2 (1–2)	2 (1–4)	4 (2–7)	< 0.001
Tumor response				
CR + PR	332 (78.9%)	57 (32.4%)	616 (83.4)	< 0.001
SD + PD	89 (21.1%)	119 (67.6%)	123 (16.6)	

*Abbreviations*: *SNUH* Seoul National University Hospital, *EUMC* Ewha Womans University Medical Center, *SMC* Samsung Medical Center, *HBV* hepatitis B virus, *HCV* hepatitis C virus, *BCLC* Barcelona Clinic Liver Cancer, *ALBI* albumin-bilirubin grade, *HAP score* hepatoma arterial-embolization prognostic score, *WBC* white blood cell, *INR* international normalized ratio, *AST* aspartate aminotransferase, *ALT* alanine aminotransferase, *NLR* neutrophil-lymphocyte ratio, *TACE* transarterial chemoembolization, *CR* complete response, *PR* partial response, *SD* stable disease, *PD* progressive disease

^a^Chronic hepatitis without cirrhosis was classified as Child-Pugh class A

There were no significant differences between the two hospital cohorts (SNUH and EUMC) with respect to sex, etiology, Child-Pugh class, tumor number, tumor size, and AFP. However, the patients in the EUMC cohort were significantly older (69 [59–78] vs. 63 [54–71] years) and had higher ALBI grades (grade 2: 67.0% vs. 58.2%), HAP scores (score D: 13.1% vs. 5.7%), and modified HAP scores (score D: 5.1% vs. 1.2%) than those in the SNUH cohort (Supplementary Table 1). Thus, instead of using each hospital cohort as either the derivation or the internal validation set, the two hospital cohorts were divided into two groups in a proportion of 7:3, which were stratified for age and sex to minimize the influence of discrepancies between the two hospital cohorts. Following the division into two sets, the discrepancies in baseline characteristics largely diminished between the derivation and internal validation sets, as shown in Table 2. Baseline characteristics of patients from SMC were also shown in Table 1. The patients of SMC were analyzed as an external validation set.

**Table 2:** Baseline characteristics of the derivation and internal validation subjects

Variables	Total (***n*** = 597)	Derivation (n = 419)	Internal validation (n = 178)	***P***
Sex				
Male	474 (79.4)	336 (80.2)	138 (77.5)	0.462
Female	123 (20.6)	83 (19.8)	40 (22.5)	
Age (year)	63.9 ± 11.7	63.9 ± 11.8	64.1 ± 11.6	0.910
Etiology				
HBV	386 (64.7)	329 (78.6)	113 (71.1)	0.868
HCV	82 (13.7)	62 (14.9)	26 (16.4)	
alcohol	60 (10.1)	28 (6.5)	20 (12.5)	
Cirrhosis				
Yes	470 (78.7%)	336 (79.8%)	134 (76.1%)	0.317
Child-Pugh class^a^				
A	479 (80.2)	340 (81.1)	139 (78.1)	0.391
B	118 (19.8)	79 (18.9)	39 (21.9)	
BCLC stage				
A	237 (39.7)	159 (37.9)	78 (43.8)	0.180
B	360 (60.3)	260 (62.1)	100 (56.2)	
ALBI grade				
1	204 (34.2)	146 (34.8)	58 (32.6)	0.046
2	363 (60.8)	258 (61.6)	105 (59.0)	
3	30 (5.03)	15 (3.6)	15 (8.4)	
HAP score				
0	4 (0.7)	3 (0.7)	1 (0.6)	0.776
A	211 (35.3)	144 (34.4)	67 (37.6)	
B	181 (30.3)	132 (31.5)	49 (27.5)	
C	154 (25.8)	105 (25.1)	49 (27.5)	
D	47 (7.9)	35 (8.4)	12 (6.7)	
Modified HAP score				
0	4 (0.7)	3 (0.7)	1 (0.6)	0.847
A	278 (46.6)	189 (45.1)	89 (50.0)	
B	217 (36.3)	155 (37.0)	62 (34.8)	
C	84 (14.1)	62 (14.8)	22 (12.4)	
D	14 (2.3)	10 (2.4)	4 (2.2)	
Tumor number				
≤3	492 (82.6)	343 (82.1)	149 (83.7)	0.713
> 3	104 (17.4)	75 (17.9)	29 (16.3)	
Tumor size (cm)				
< 3	247 (41.4)	169 (40.3)	78 (43.8)	0.725
3–4	180 (30.1)	128 (30.5)	52 (29.2)	
≥5	170 (28.5)	122 (29.1)	48 (27.0)	
Hemoglobin (g/dL)	12.9 ± 2.1	13.0 ± 2.0	12.6 ± 2.2	0.08
WBC (×10^3^/μL)	5.10 (3.9–6.3)	5.18 (3.9–6.4)	5.01 (3.8–6.1)	0.362
Platelet (×10^3^/μL)	128 (83–170)	131 (84–176)	115.5 (79–160)	0.038
Prothrombin time (INR)	1.1 (1.0–1.2)	1.1 (1.0–1.2)	1.1 (1.0–1.2)	0.133
Creatinine (mg/dL)	0.9 (0.7–1.0)	0.9 (0.8–1.0)	0.9 (0.7–1.0)	0.099
Sodium (mEq/L)	138.9 ± 3.1	139.0 ± 3.2	138.7 ± 3.0	0.377
AST (mg/dL)	42 (30–64)	42 (30–65)	42 (30–60)	0.627
ALT (mg/dL)	33 (22–54)	34 (23–55)	31 (21–54)	0.255
Total bilirubin (mg/dL)	0.8 (0.6–1.2)	0.8 (0.6–1.1)	0.8 (0.6–1.2)	0.383
Albumin (g/dl)	3.8 (3.4–4.1)	3.8 (3.4–4.0)	3.7(3.4–4.1)	0.158
Alpha-fetoprotein (ng/mL)				
≥200	138 (23.3)	103 (24.8)	35 (19.8)	0.189
< 200	455 (76.7)	313 (75.2)	142 (80.2)	
NLR	1.87 (1.3–2.7)	1.85 (1.3–2.6)	1.95 (1.3–2.8)	0.412
No. of TACE sessions	2.9 ± 2.8	3.0 ± 2.8	2.9 ± 2.9	0.290
Tumor response				
CR + PR	389 (65.2%)	273 (65.2%)	116 (65.2%)	0.997
SD + PD	208 (34.8%)	146 (34.8%)	62 (34.8%)	

*Abbreviations*: *HBV* hepatitis B virus, *HCV* hepatitis C virus, *BCLC* Barcelona Clinic Liver Cancer, *ALBI* albumin-bilirubin grade, *HAP score* hepatoma arterial-embolization prognostic score, *WBC* white blood cell, *INR* international normalized ratio, *AST* aspartate aminotransferase, *ALT* alanine aminotransferase, *NLR* neutrophil-lymphocyte ratio, *TACE* transarterial chemoembolization, *CR* complete response, *PR* partial response, *SD* stable disease, *PD* progressive disease

^a^Chronic hepatitis without cirrhosis was classified as Child-Pugh class A

### Identification of prognostic factors of OS after initial TACE treatment

The Cox proportional hazards analyses were performed in the derivation set. Univariate Cox analysis was performed to select prognostic factors associated with OS, and the following variables were significantly associated with OS: Child-Pugh class B, AFP ≥200 ng/mL, ALBI grade 2, tumor number ≥ 3, maximal tumor diameter ≥ 5 cm, initial TACE response, and HAP score D. Multivariate analysis identified four independent predictors for OS as follows: ALBI grade (adjusted hazard ratio [aHR], 2.299; 95% CI, 1.694–3.119; *P <* 0.001), maximal tumor diameter (aHR, 1.704; 95% CI, 1.424–2.038; *P <* 0.001), AFP (aHR, 1.583; 95% CI, 1.164–2.154; *P* = 0.003), and initial TACE response (aHR, 1.915; 95% CI, 1.431–2.563; *P <* 0.001) (Table 3).

**Table 3:** Uni- and multivariate analysis for overall survival in the derivation cohort

Variables	Univariate analysis			Regression coefficient ***(ß)***		Multivariate analysis	
	HR (95% CI)		***P***			HR (95% CI)	***P***
Sex							
Female	0.940	(0.662–1.335)	0.731				
Age (year)	1.024	(1.012–1.037)	< 0.001				
Etiology							
Non-Viral	ref						
Viral	0.616	(0.437–0.870)	0.006				
Child-Pugh class							
A	ref						
B	1.591	(1.143–2.213)	0.006				
ALBI grade							
1	ref						
2	2.231	(1.605–3.101)	<.0001				
3	6.602	(3.409–12.786)	<.0001	0.832	2.299	(1.694–3.119)	<.0001
HAP score							
A-C	ref						
D	3.937	(2.872–5.399)	<.0001				
Modified HAP score							
A-C	ref						
D	4.689	(3.012–7.300)	<.0001				
Tumor number							
≤3	ref						
> 3	1.615	(1.155–2.259)	0.005				
Tumor size (cm)							
< 3	ref						
3–4	1.775	(1.231–2.560)	0.002				
≥5	3.359	(2.368–4.763)	<.0001	0.533	1.704	(1.424–2.038)	<.0001
Hemoglobin (g/dL)	0.851	(0.796–0.909)	<.0001				
Log WBC	1.452	(0.988–2.132)	0.058				
Log platelet	1.165	(0.875–1.551)	0.296				
Log prothrombin time (INR)	9.847	(3.454–28.076)	<.0001				
Log creatinine	1.907	(1.377–2.640)	<.0001				
Sodium	0.937	(0.898–0.977)	0.002				
Log AST	2.332	(1.833–2.966)	<.0001				
Log ALT	1.231	(1.010–1.499)	0.039				
Log Total bilirubin	1.191	(0.900–1.574)	0.221				
Alpha-fetoprotein							
< 200	ref						
≥200	1.774	(1.313–2.397)	<.0001	0.460	1.583	(1.164–2.154)	0.0034
NLR	1.91	(1.444–2.525)	<.0001				
Tumor response							
CR + PR	ref						
SD + PD	2.292	(1.733–3.032)	<.0001	0.650	1.915	(1.431–2.563)	<.0001

*Abbreviations*: *HR* hazards ratio, *ref.* reference, *ALBI* albumin-bilirubin grade, *HAP score* hepatoma arterial-embolization prognostic score, *INR* international normalized ratio, *AST* aspartate aminotransferase, *ALT* alanine aminotransferase, *NLR* neutrophil-lymphocyte ratio, *CR* complete response, *PR* partial response, *SD* stable disease, *PD* progressive disease

### Prediction model development for evaluating TACE suitability

Through forward stepwise selection, a scoring system was developed including the aforementioned four independent predictors for OS (i.e., ALBI, tumor size, AFP, initial TACE response; abbreviated as “ASAR”) as shown in Table 4. Regression coefficients of the factors were 0.832 (ALBI grade), 0.533 (maximal tumor diameter), 0.460 (AFP), and 0.650 (initial TACE response). The weighted scores (0, 1, and 2) for the ASAR scoring system were assigned for the four covariates based on the regression coefficients that were obtained from the final analysis and the total scores ranged from 0 to 6. We confirmed the simplest model according to the result of the Hosmer-Lemeshow test and c-statistics between models with the abovementioned statistical methodology. C-index for OS was 0.733 (95% CI, 0.570–0.871) in the derivation set, which was maintained at 0.733 (95% CI, 0.703–0.768) with 100-fold bootstrapping. In the internal validation, the c-index was 0.700 (95% CI, 0.445–0.905). Goodness-of-fit for the ASAR model was confirmed in both the derivation and the internal validation sets (*P* = 0.360 and *P* = 0.926, respectively, by Hosmer-Lemeshow χ^2^ test). In the external validation set, c-index was maintained at 0.680 (95% CI, 0.652–0.707).

**Table 4:** ASAR (ALBI-size-AFP-response) scoring system

		Score	
	0	1	2
ALBI grade	1	2	3
Maximal tumor diameter (cm)	< 3	3–5	≥5
AFP (ng/mL)	< 200	≥200	
Tumor response	CR, PR	SD, PD	

*Abbreviations*: *ALBI* albumin-bilirubin grade, *AFP* alpha-fetoprotein, *CR* complete response, *PR* partial response, *SD* stable disease, *PD* progressive disease

**Fig. 2:**
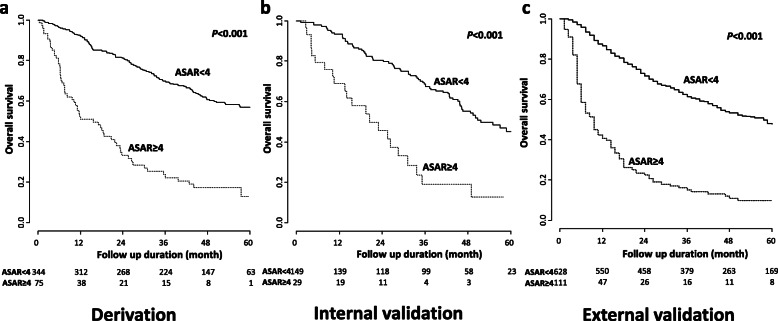
(a-c) Survival analyses of the derivation and validation set according to ASAR score. ASAR scores (cut-off = 4) offered similar predictive performance of overall survival in the internal and external validation set compared to that in the derivation set

Based on the pairwise log-rank test results, the cut-off value was designated as 4, which presented the greatest difference in the survival curves of the derivation set. In the derivation set, patients with ASAR < 4 had significantly longer OS than ASAR ≥4 (hazard ratio [HR], 0.233; 95% CI, 0.171–0.317; *P <* 0.001; Fig. 2a). In the internal validation set, patients with ASAR < 4 had significantly longer OS than ASAR ≥4 (HR, 0.287; 95% CI, 0.176–0.468; *P <* 0.001; Fig. 2b). Thus, we designated ASAR < 4 as the low-risk group and ASAR ≥4 as the high-risk group. Among 178 patients of the internal validation set, the proportion of patients who were not amenable to further treatment after initial TACE was higher in the high-risk group (4 out of 29, 13.8%) than in the low-risk group (5 out of 149, 3.4%) (*P* = 0.019). Of these nine patients, six patients died after first TACE and three patients received best supportive care. We also compared ASAR with ART score for the decision of TACE repetition, and ASAR showed better performance than ART score in the internal validation set (Supplementary Table 2). In the external validation set, patients with ASAR < 4 had also significantly longer OS than ASAR ≥4 (HR, 0.237; 95% CI, 0.189–0.298; *P <* 0.001; Fig. 2c).

### Sensitivity analyses

**Fig. 3:**
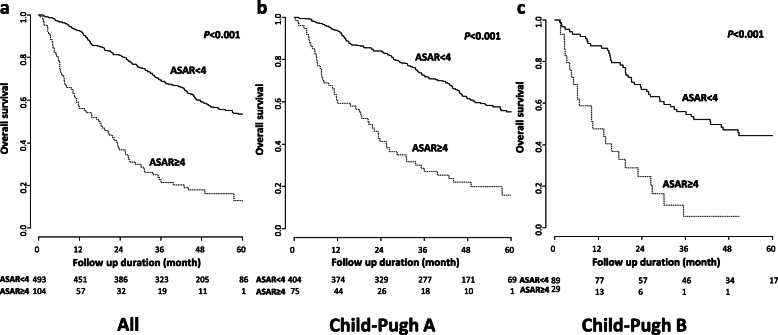
(a-c) Survival analyses of subgroups according to Child-Pugh class with ASAR score in the entire cohort. When applying ASAR score (cut-off = 4), overall survivals were significantly different in all enrolled patients as well as in patients with Child-Pugh class A or B

In the combined derivation and internal validation cohorts (SNUH and EUMC), the low-risk group showed significantly longer OS than that shown by the high-risk group (HR, 0.249; 95% CI, 0.192–0.324; *P <* 0.001; Fig. 3a). The median OS was 70.2 months in the low-risk group vs. 17.7 months in the high-risk group. The 1-year and 3-year survival rates were 92.1 and 69.7%, respectively, in the low-risk group and 52.4 and 23.7%, respectively, in the high-risk group. Among a subgroup of patients with Child-Pugh class A, the low-risk group showed significantly longer OS than the high-risk group (HR, 0.260; 95% CI, 0.191–0.354; *P <* 0.001; Fig. 3b). The 1-year and 3-year survival rates were 93.4 and 72.4% in the low-risk group, and 59.4 and 27.0% in the high-risk group, respectively. Among the subgroup of patients with Child-Pugh class B, the low-risk group showed significantly longer OS than that shown by the high-risk group (HR, 0.252; 95% CI, 0.151–0.420; *P <* 0.001; Fig. 3c). The 1-year and 3-year survival rates were 85.2 and 53.4% in the low-risk group, and 46.3 and 6.6% in the high-risk group, respectively.

Survivals from the subanalysis for BCLC-stage B patients were also comparable to those of the entire patients. The low-risk group showed significantly longer OS than the high-risk group both in the derivation (HR, 0.293; 95% CI, 0.190–0.452; *P* < 0.001; Supplementary Fig. 1a) and internal validation sets (HR, 0.261; 95% CI, 0.132–0.516; *P* < 0.001; Supplementary Fig. 1b). The 1-year and 3-year survival rates of derivation set were 88.6 and 64.1% in the low-risk group, and 46.3 and 6.6% in the high-risk group, respectively. The 1-year and 3-year survival rates of validation set were 86.7 and 65.3%, in the low-risk group, and 67.3 and 14.2% in the high-risk group, respectively.

### Survival prediction in child-Pugh class B patients before the initial TACE

**Fig. 4:**
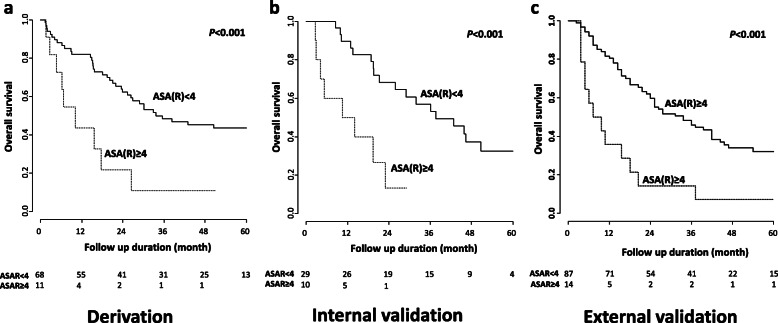
(a-c) Overall survival in patients with Child-Pugh class B according to the modified model without initial TACE response (ALBI, size of tumor, and AFP) in the derivation and validation sets. When applying the modified model (cut-off = 4) score only in patients with Child-Pugh class B, overall survivals were significantly different in patients in the derivation and the internal and external validation sets

Because the initiation of first TACE is an important issue for patients with Child-Pugh class B, we additionally assessed the predictive performance for those patients using a modified version of the ASAR scoring system, which comprised of only baseline components (ALBI, size, and AFP) excluding initial TACE response, i.e., “ASA(R)”. C-index values were 0.788 (95% CI, 0.702–0.876) in the derivation set, 0.745 (95% CI, 0.646–0.862) in the internal validation set, and 0.670 (95% CI, 0.605–0.725) in the external validation set, respectively. Using the cut-off score of 4 for this modified ASA(R) in Child-Pugh class B patients, OS was also significantly different among the derivation set (ASA(R) < 4 vs. ≥4: HR, 0.317; 95% CI, 0.150–0.669; *P <* 0.001; Fig. 4a), the internal validation set (ASA(R) < 4 vs. ≥4: HR, 0.225; 95% CI, 0.088–0.575; *P <* 0.001; Fig. 4b), and the external validation set (ASA(R) < 4 vs. ≥4: HR, 0.261; 95% CI, 0.201–0.339; *P <* 0.001; Fig. 4c). ASA(R) showed significantly better performance than other models for evaluating initial TACE suitability, such as HAP, modified HAP, and modified HAP II models in the internal validation set (Supplementary Table 2).

## Discussion

The prediction model (ASAR) of the present study seems easily applicable for HCC patients undergoing TACE in practice and may be helpful in decision making on TACE repetition. This prediction model incorporated objective assessment of baseline hepatic functional reserve as well as tumor burden and treatment response, achieving better predictive performance compared to other prediction models. In addition, the modification of this model using only baseline factors also showed meaningful predictive value in patients with Child-Pugh class B, which could particularly be helpful for the decision on the initiation of TACE.

Although TACE is the standard-of-care for patients with intermediate-stage HCC (BCLC stage B), these patients encompass a wide range of the disease spectrum [25]. Thus, several prediction models have been developed to select more suitable patients for TACE, such as ART score, ABCR (AFP, BCLC, Child-Pugh score, and response) score, (modified) HAP score, and STATE-score [7, 9, 25, 26]. However, it seems premature to widely apply these models in practice given that they have not shown consistent results in subsequent studies [11, 27].

When designing the present study, we raised two practical questions regarding the decision for TACE. The first one was “when to stop TACE?” in patients with good hepatic function who proceed to first TACE with less concerns on the potential deterioration of hepatic function (i.e., mostly Child-Pugh class A). For those patients, early prediction of TACE failure or refractoriness seems more relevant, in order to switch to systemic therapy before their hepatic functions deteriorate with futile repetition of TACE [21, 28]. The other question was “whether to proceed to first TACE or not” in patients with impaired hepatic functional reserve at the time of HCC diagnosis. Although its use is not generally encouraged for these patients, TACE sometimes becomes the only treatment option, particularly when tumor burden exceeds the criteria for liver transplantation or when transplantation is not readily feasible due to organ shortage. Systemic therapy also has not been proved to be effective in these patients [2, 3, 29]. Hence, cautious application of TACE for properly selected patients with Child-Pugh score ≤ B8 might be beneficial for these patients, given that Child-Pugh score > 8 is generally regarded as a contraindication for TACE [30].

The prediction model of this study includes four relevant risk factors, namely, ALBI grade, maximal tumor size, baseline AFP, and initial TACE response. For hepatic functional assessment, especially in HCC patients, ALBI grade has proved to be useful in previous studies [14] as well as in our cohorts. The conventional Child-Pugh scoring system has been used widely as a standard method for the assessment of hepatic function in HCC patients until now. However, the Child-Pugh system includes subjective (due to grade of ascites and hepatic encephalopathy) and interrelated (i.e., albumin and ascites) components, and has no weighting scores on each component [13]. The recently developed ALBI grade offers a simple, objective, and discriminatory method of assessing hepatic function in patients with HCC [16]. Because the Child-Pugh system is still used as a primary measure for the selection of treatment options in major HCC guidelines [2, 3], we adopted a step-wise approach in this study. First, because TACE is recommended for Child-Pugh class A and highly selected class B, such patients were first selected (i.e., ‘entire cohort’) and the prediction model was developed. Then, the predictive performance of the model was evaluated in Child-Pugh class A and B patients separately. While our model originally expresses an adaptive strategy using baseline characteristics plus initial TACE response to provide information on when to stop TACE, the modified model which only includes the baseline factors, i.e., “ASA(R)”, well discriminated the prognostic subgroups for Child-Pugh class B patients, suggesting the usefulness in selecting patient suitability for first TACE in patients with impaired hepatic function.

Tumor size and AFP level reflect tumor burden in our model, and were also included in previous prediction models such as ABCR and HAP score [8, 31]. Although tumor number was also a relevant prognostic factor in the multivariable analysis, it was excluded in our final prediction model from the stepwise selection procedure for the model derivation. Some previous models also excluded tumor number, for example, models for the decision of first TACE (HAP, modified HAP) and ART score (for TACE repetition) [7–9]. However, other models included tumor number, such as the STATE score, modified HAP-II score, and “six-and-twelve” score (all for the decision of first TACE) or SNACOR (for TACE repetition) [26, 32–34]. Tumor size is a well-known factor for determining the achievement of objective response following TACE [35]. Moreover, because the majority of the study population in the entire cohort had 3 or less tumors, the relative relevance of tumor number might have been offset in the derivation of the model.

TACE response has been included in previous models for decision making on TACE repetition [7, 31, 32] as well as in consensus papers or guidelines defining TACE refractoriness [28, 36–38]. TACE failure or refractoriness in the literature mostly requires two consecutive absences of objective response, which is not supported by solid evidences. In the present study, we assessed the relevance of initial TACE response and incorporated it as a component of the model, given that an initial TACE response was the most robust predictor for the outcome in a recent study [39]. Furthermore, recent advances in systemic treatment options for HCC, such as second-line agents or immunotherapy, appear to facilitate earlier decision on whether to repeat TACE or switch to systemic therapy [40, 41]. The concept of treatment stage migration or switching to a systemic agent such as sorafenib might be of little benefit once hepatic functional deterioration develops with repeated TACE [29]. Given that earlier prediction of TACE failure was the fundamental goal of the present study, on-treatment hepatic functional deterioration, such as increase in the Child-Pugh score, was not taken into consideration for model derivation, because further treatment would quite be limited with Child-Pugh score increase under repeated TACE. Instead, our model was developed to predict TACE failure earlier using the initial TACE response in Child-Pugh class A patients, thereby enabling an earlier switch to systemic treatment, and to prevent harmful results of TACE initiation in Child-Pugh class B patients using solely baseline characteristics as described earlier.

The strengths of the prediction model in the present study comprise its simplicity and good performance, which was derived from a relatively large study population from institutions with plenty of experience in TACE and technical similarity in the TACE procedure. Moreover, the performance of this model showed reproducibility in both internal and external validations. However, there are several limitations in the present study. First, tumor number may be a relevant component for the prediction model as shown in previous studies, although it was not included in the stepwise selection for model derivation in the present study. In addition, relevance of tumor markers other than AFP, particularly protein induced by vitamin K antagonist-II, was not evaluable because of incomplete data. These potentially relevant tumor-related factors need to be evaluated for the prediction model. Lastly, although this was a multi-institutional study including a relatively large number of patients, model derivation and validation were conducted in a combined set of two institutional cohorts (SNUH, EUMC) instead of using each institutional cohort as either derivation or validation set, because several baseline characteristics were significantly different between the two cohorts. At least, however, our model was equally discriminatory when applied to each institutional cohort separately and we also validated the model externally in an independent cohort.

## Conclusion

In conclusion, a simple scoring system (ASAR) may be helpful for decision making on the repetition and/or initiation of TACE in patient with unresectable HCC. This prediction model could be applied differently according to patients’ baseline hepatic functional reserve, that is, an earlier switch or treatment stage migration based on the score with the initial TACE response for patients with good baseline hepatic function versus decision on whether to implement first TACE or not for patients with impaired hepatic function based on baseline factors only. The former strategy could lead patients to systemic therapy prior to further deterioration of hepatic function, and the latter could help patients avoid potentially harmful treatment.

## Supplementary information

**Additional file 1 Supplementary Fig. 1** (a, b) Survival analyses of derivation and validation set according to ASAR score in patients with BCLC-B ASAR scores (cut-off = 4) offered similar predictive performance of overall survival in the validation set compared to that in the derivation set in patients with BCLC-B. **Supplementary Fig. 2** (a, b) Comparison of overall survival in patients with Child-Pugh B according to HAP, and mHAP in validation set. Between high and low risk group according to HAP and modified HAP score, overall survivals were not significantly different.

**Additional file 2 Supplementary Table 1**. Baseline characteristics of the SNUH and EUMC cohort **Supplementary Table 2**. Comparison of *c*-indices among models in internal validation cohort **Supplementary material.**

## Data Availability

The datasets used and/or analysed during the current study are available from the corresponding author on reasonable request.

## Abbreviations

HCC: Hepatocellular carcinoma
TACE: Transarterial chemoembolization
BCLC: Barcelona Clinic Liver Cancer
HAP: Hepatoma arterial-embolization prognostic
ART: Assessment for retreatment with TACE
ALBI: Albumin-bilirubin
SNUH: Seoul National University Hospital
EUMC: Ewha Womans University Medical Center
OS: Overall survival
IQR: Interquartile ranges
AFP: Alpha-fetoprotein
