# Supplementary material for: A differential risk assessment and decision model for Transarterial chemoembolization in hepatocellular carcinoma based on hepatic function
Source: BMC Cancer. 2020 Jun 1;20:504. doi: 10.1186/s12885-020-06975-2 (PMC7268402; doi:10.1186/s12885-020-06975-2)
Supplement: Supplementary file 1 — Additional file 1 Supplementary Fig. 1 (a, b) Survival analyses of derivation and validation set according to ASAR score in patients with BCLC-B ASAR scores (cut-off = 4) offered similar predictive performance of overall survival in the validation set compared to that in the derivation set in patients with BCLC-B. Supplementary Fig. 2 (a, b) Comparison of overall survival in patients with Child-Pugh B according to HAP, and mHAP in validation set. Between high and low risk group according to HAP and modified HAP score, overall survivals were not significantly different. [file 12885_2020_6975_MOESM1_ESM.pptx]

## Slide 1
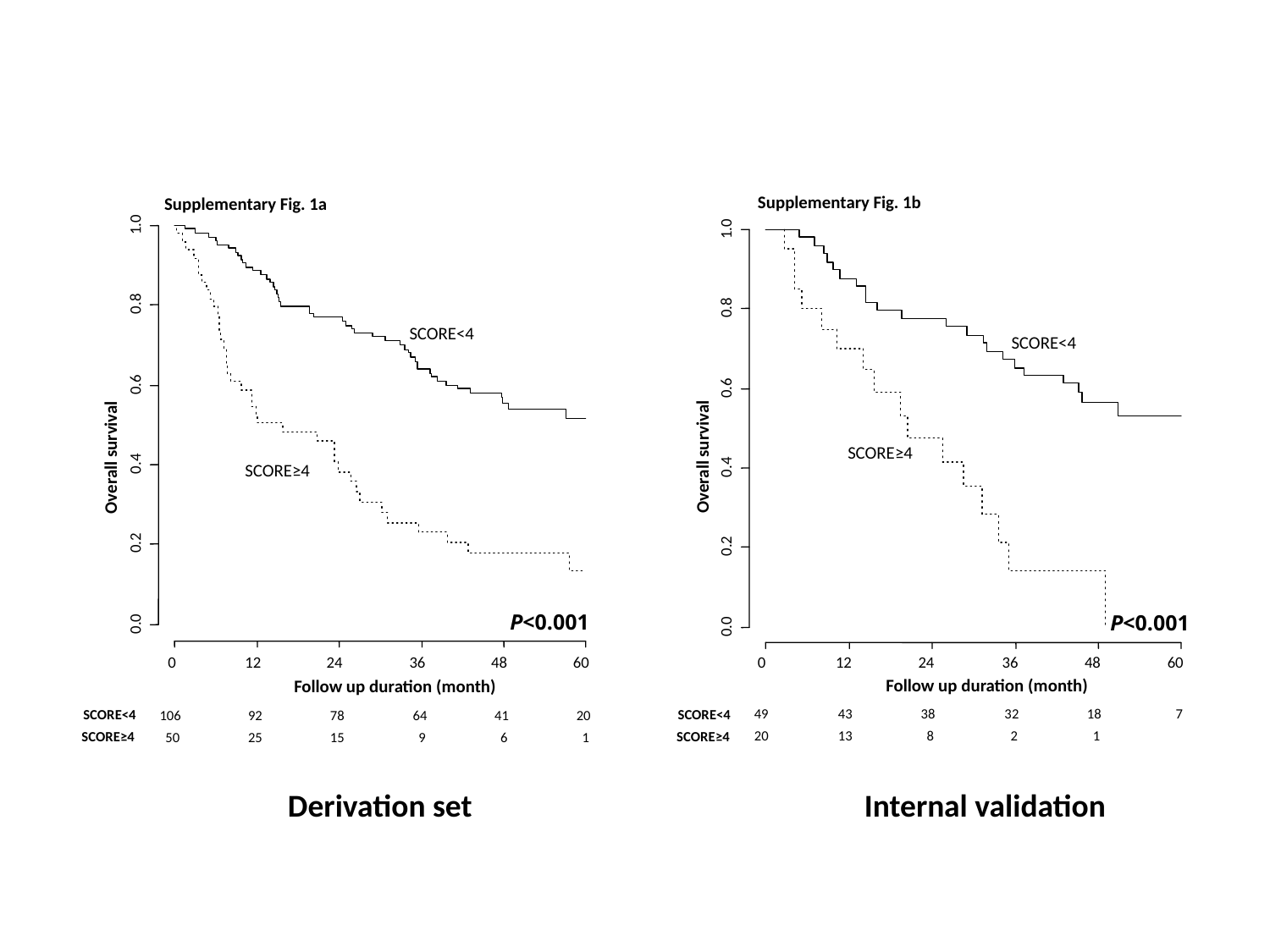

Supplementary Fig. 1b
Supplementary Fig. 1a
1.0
1.0
0.8
0.8
SCORE<4
SCORE<4
0.6
0.6
Overall survival
Overall survival
SCORE≥4
0.4
0.4
SCORE≥4
0.2
0.2
P<0.001
P<0.001
0.0
0.0
0
12
24
36
48
60
0
12
24
36
48
60
Follow up duration (month)
Follow up duration (month)
49
43
38
32
18
7
SCORE<4
SCORE<4
106
92
78
64
41
20
50
25
15
9
6
1
20
13
8
2
1
SCORE≥4
SCORE≥4
Derivation set
Internal validation

## Slide 2
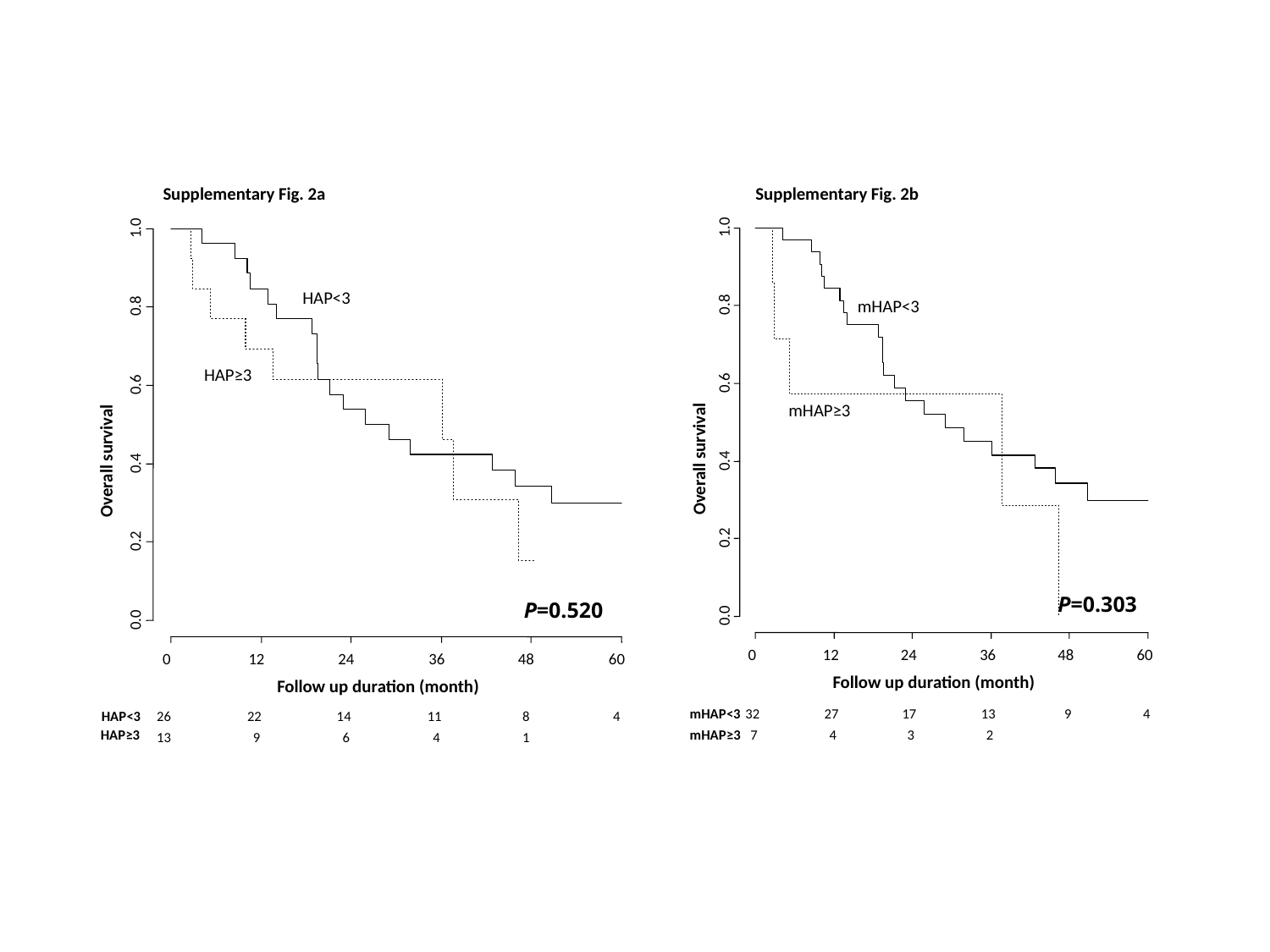

Supplementary Fig. 2a
Supplementary Fig. 2b
1.0
1.0
HAP<3
0.8
0.8
mHAP<3
HAP≥3
0.6
0.6
mHAP≥3
Overall survival
Overall survival
0.4
0.4
0.2
0.2
P=0.303
P=0.520
0.0
0.0
0
12
24
36
48
60
0
12
24
36
48
60
Follow up duration (month)
Follow up duration (month)
32
27
17
13
9
4
mHAP<3
HAP<3
26
22
14
11
8
4
HAP≥3
7
4
3
2
mHAP≥3
13
9
6
4
1
