# Supplementary material for: A differential risk assessment and decision model for Transarterial chemoembolization in hepatocellular carcinoma based on hepatic function
Source: BMC Cancer. 2020 Jun 1;20:504. doi: 10.1186/s12885-020-06975-2 (PMC7268402; doi:10.1186/s12885-020-06975-2)
Supplement: Supplementary file 2 — Additional file 2 Supplementary Table 1. Baseline characteristics of the SNUH and EUMC cohort Supplementary Table 2. Comparison of c-indices among models in internal validation cohort Supplementary material. [file 12885_2020_6975_MOESM2_ESM.docx]

**Supplementary table 1**. Baseline characteristics of the SNUH and EUMC cohort

| **Variables** |  | **Total (n=597)** | **SNUH (n=421)** | **EUMC (n=176)** | ***P*** |
| --- | --- | --- | --- | --- | --- |
| Sex | Male | 474 (79.4) | 333 (79.1) | 141 (80.1) | 0.780 |
|  | Female | 123 (20.6) | 88 (20.9) | 35 (19.9) |  |
| Age (year) |  | 64 (56-73) | 63 (54-71) | 69 (59-78) | <0.001 |
| Etiology | HBV | 386 (64.7) | 265 (63.0) | 121 (74.6) | 0.238 |
|  | HCV | 82 (13.7) | 56 (13.3) | 26 (16.0) |  |
|  | alcohol | 60 (10.1) | 45 (10.7) | 15 (9.4) |  |
| Cirrhosis | Yes | 470 (78.7%) | 336 (79.8%) | 134 (76.1%) | 0.317 |
| Child-Pugh class† | A | 479 (80.2) | 336 (79.8) | 143 (81.3) | 0.687 |
|  | B | 118 (19.8) | 85 (20.2) | 33 (18.8) |  |
| BCLC stage | A | 237 (39.7) | 168 (39.9) | 69 (39.2) | 0.873 |
|  | B | 360 (60.3) | 253 (60.1) | 107 (60.8) |  |
| ALBI grade | 1 | 204 (34.2) | 159 (37.8) | 45 (25.6) | 0.008 |
|  | 2 | 363 (60.8) | 245 (58.2) | 118 (67.0) |  |
|  | 3 | 30 (5.3) | 17 (4.0) | 13 (7.4) |  |
| HAP score | 0 | 4 (0.7) | 0 (0.0) | 4 (2.3) | <0.001 |
|  | A | 211 (35.3) | 157 (37.3) | 54 (30.7) |  |
|  | B | 181 (30.3) | 134 (31.8) | 47 (26.7) |  |
|  | C | 154 (25.8) | 106 (25.2) | 48 (27.3) |  |
|  | D | 47 (7.9) | 24 (5.7) | 23 (13.1) |  |
| Modified HAP score | 0 | 4 (0.7) | 0 (0.0) | 4 (2.3) | 0.001 |
|  | A | 278 (46.6) | 211 (50.1) | 67 (38.1) |  |
|  | B | 217 (36.3) | 150 (35.6) | 67 (38.1) |  |
|  | C | 84 (14.1) | 55 (13.1) | 29 (16.5) |  |
|  | D | 14 (2.3) | 5 (1.2) | 9 (5.1) |  |
| Tumor number | ≤3 | 492 (82.6) | 349 (82.9) | 143 (81.7) | 0.819 |
|  | >3 | 104 (17.4) | 72 (17.1) | 32 (18.3) |  |
| Tumor size (cm) | <3 | 247 (41.4) | 179 (42.5) | 68 (38.6) | 0.386 |
|  | 3-4 | 180 (30.1) | 129 (30.6) | 51 (29.0) |  |
|  | ≥5 | 170 (28.5) | 113 (26.8) | 57 (32.4) |  |
| Hemoglobin (g/dL) |  | 13.1 (11.7-14.3) | 13.0 (11.8-14.3) | 13.2 (11.3-14.5) | <0.001 |
| WBC (x10³/µL) |  | 5.10 (3.9-6.3) | 5.10 (3.9-6.3) | 5.10 (3.9-6.3) | 0.733 |
| Platelet (x10³/µL) |  | 128 (83-170) | 120 (81-165) | 132 (91-179) | 0.072 |
| Prothrombin time (INR) |  | 1.1 (1.0-1.2) | 1.1 (1.0-1.2) | 1.1 (1.1-1.2) | <0.001 |
| Creatinine (mg/dL) |  | 0.9 (0.7-1.0) | 0.8 (0.7-1.0) | 0.9 (0.8-1.1) |  |
| Sodium (mEq/L) |  | 139 (137-141) | 141 (139-142) | 139 (137-141) | 0.001 |
| AST (mg/dL) |  | 42 (30-64) | 42 (29-64) | 44 (33-63.5) | 0.125 |
| ALT (mg/dL) |  | 33 (22-54) | 34 (22-54) | 33 (23-53.5) | 0.990 |
| Total bilirubin (mg/dL) |  | 0.8 (0.6-1.2) | 0.8 (0.6-1.2) | 0.8 (0.6-1.1) | 0.254 |
| Alpha-fetoprotein (ng/mL) | ≥200 | 138 (23.3) | 89 (21.1) | 49 (28.5) | 0.055 |
|  | <200 | 455 (76.7) | 332 (78.9) | 123 (71.5) |  |
| NLR |  | 1.87 (1.3-2.7) | 1.85 (1.3-2.6) | 1.90 (1.3-3.1) | 0.343 |
| Number of TACE sessions |  | 2 (1-3) | 2 (1-2) | 2 (1-4) | 0.246 |
| Tumor response | CR+PR | 389 (65.2%) | 332 (78.9%) | 57 (32.4%) | <0.001 |
|  | SD+PD | 208 (34.8%) | 89 (21.1%) | 119 (67.6%) |  |

Abbreviations: SNUH, Seoul National University Hospital; EUMC, Ewha Womans University Medical Center; HBV, hepatitis B virus; HCV, hepatitis C virus; BCLC, Barcelona Clinic Liver Cancer; ALBI, albumin-bilirubin grade; HAP score, hepatoma arterial-embolization prognostic score; WBC, white blood cell; INR, international normalized ratio; AST, aspartate aminotransferase; ALT, alanine aminotransferase; NLR, Neutrophil-Lymphocyte ratio; TACE, transarterial chemoembolization; CR, complete response; PR, partial response; SD, stable disease; PD, progressive disease.

†Chronic hepatitis without cirrhosis was classified as Child-Pugh class A.

**Supplementary table 2**. Comparison of *c*-indices among models in internal validation cohort

| **Model** | ***c*-index** | **95% confidence interval** | | ***P*-value** |
| --- | --- | --- | --- | --- |
|  |  | **Lower** | **Upper** |  |
| ASAR | 0.700 | 0.445 | 0.905 | Ref 1. |
| ART | 0.656 | 0.542 | 0.770 | 0.016**^†^** |
| ASA(R) | 0.745 | 0.646 | 0.862 | Ref 2. |
| HAP | 0.618 | 0.553 | 0.683 | <0.001^‡^ |
| Modified HAP | 0.538 | 0.498 | 0.578 | <0.001^‡^ |
| Modified HAP II | 0.600 | 0.524 | 0.676 | <0.001^‡^ |

†Compared to the *c*-index of the ASAR

‡Compared to the *c*-index of the ASA(R)

**Supplementary material**

1) Hepatoma arterial-embolization prognostic (HAP) score

| **Prognostic factor** | **Points** |
| --- | --- |
| Albumin < 36 g/dL | 1 |
| Alpha-fetoprotein > 400 ng/mL | 1 |
| Bilirubin > 17 umol/L | 1 |
| Maximum tumor diameter > 7cm | 1 |
| **HAP classification** | **Points** |
| HAP A | 0 |
| HAP B | 1 |
| HAP C | 2 |
| HAP D | >2 |

2) Albumin-bilirubin (ALBI) score

= (log_10_ bilirubin [μmol/L] x 0.66) + (albumin [g/L] x -0.085)

| **ALBI grade:** |  |
| --- | --- |
| Grade 1 | ALBI score ≤ -2.60 |
| Grade 2 | -2.60 < ALBI score ≤ -1.39 |
| Grade 3 | -1.39 < ALBI score |

**Supplementary Figure Legends**

**Supplementary Fig. 1 (a, b) Survival analyses of derivation and validation set according to ASAR score in patients with BCLC-B**

ASAR scores (cut-off=4) offered similar predictive performance of overall survival in the validation set compared to that in the derivation set in patients with BCLC-B.

**Supplementary Fig. 2 (a, b) Comparison of overall survival in patients with Child-Pugh B according to HAP, and mHAP in validation set.**

Between high and low risk group according to HAP and modified HAP score, overall survivals were not significantly different.
